# Protocol for preparing cellulose-based aerogels in a deep eutectic solvent as surface-enhanced Raman scattering substrates

**DOI:** 10.1016/j.xpro.2025.103795

**Published:** 2025-04-25

**Authors:** Kaori Sánchez-Carrillo, Sandeep Surendra Panikar, Josué D. Mota-Morales

**Affiliations:** 1 Centro de Física Aplicada y Tecnología Avanzada, Universidad Nacional Autónoma de México, Querétaro 76230, Mexico; 2 Division of Oncology, Department of Medicine, Washington University School of Medicine, St. Louis, MO, USA

**Keywords:** Physics, Chemistry, Material sciences

## Abstract

There is an increasing need to detect and identify analytes of diverse origins in low molar concentrations and from complex matrices. Here, we introduce a protocol for preparing surface-enhanced Raman scattering (SERS) substrates that enable the detection of aqueous analytes in ultralow concentrations. We describe the procedure for synthesizing plasmonic aerogels using L-ascorbic acid as a reductor and eutectic solvents as reaction media. We then detail procedures for measurements using these substrates.

For complete details on the use and execution of this protocol, please refer to Panikar et al.^1^

## Highlights

• Steps to synthesize SERS substrates based on gold nanoparticles and cellulose
• Outlines a methodology for the synthesis in nonaqueous media
• Procedures for substrate fabrication and sample preparation

## Abstract (editor highlights)

Publisher’s note: Undertaking any experimental protocol requires adherence to local institutional guidelines for laboratory safety and ethics.

## Graphical abstract

**Figure:**
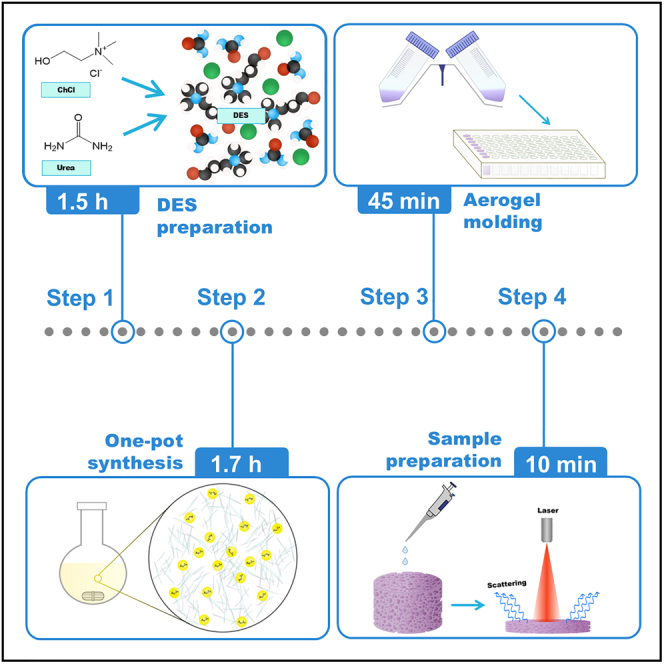

## Before you begin

Surface-enhanced Raman scattering (SERS) has gained significant attention in recent years due to its outstanding selectivity and sensitivity,^2^^,^^3^ which promises to provide efficient sensing devices for biomedical and environmental monitoring needed to face the current challenges.^3^ Studies have shown that substrates exhibiting higher uniformity and tunable distances between plasmonic nanostructures lead to optimum sites for the detection of diverse analytes.^4^^,^^5^ This phenomenon is a direct consequence of the localized surface plasmon resonance of metallic nanoparticles, which creates local maxima of the electromagnetic field when irradiated with a laser. Areas between tips, gaps or crevices of metallic nanoparticles are known as ‘hotspots’ due to the elevated amplification of the electromagnetic field, which results from the overlapping enhancement of close-packed structures.^4^^,^^6^^,^^7^

Most newly developed SERS substrates exhibit a substantial tradeoff between efficiency, uniformity, and stability.^8^ For instance, SERS substrates fabricated using lithography show high uniformity, but the cost and complex systems involved in their fabrication hinder its wide implementation.^2^^,^^9^^,^^10^ On the other hand, colloidal substrates have demonstrated the maximum SERS enhancement and sensitivity down to single-molecule detection; however, the agglomerations formed after analytes are introduced typically result in low-density hotspots for measurement.^11^^,^^12^ Therefore, there is considerable room for improvement in the development of SERS substrates, particularly toward robust and self-standing 3D composites.

Moreover, implementing sustainability strategies has become one of the main goals in applied sciences. Hence, the fabrication of SERS substrates with 3D hotspot density using sustainable strategies, such as sustainable solvents and biobased ingredients, will contribute to the development of greener technologies.^5^^,^^13^ In this context, cellulose nanocrystals (CNCs), which are derived from naturally occurring cellulose – the most abundant biopolymer – are renewable and biodegradable, making them ideal for the fabrication of substrates with a green chemistry approach.^14^ In addition, cellulose exhibits suitable chemical functionalities (e.g., −OH) for the *in-situ* reduction of metallic nanoparticle precursors.^15^^,^^16^ The combination of plasmonic nanoparticles with cellulose for the fabrication of SERS substrates has been the subject of intense research.

Furthermore, deep eutectic systems (DESs), and eutectic solvents more broadly, are a family of sustainable reaction media composed of mixtures of pairs of hydrogen acceptors and donors. They have been used as designer solvents as they allow many chemical reactions to occur in a nonaqueous environment while exhibiting unusual solvation capabilities, and tunability in terms of viscosity and polarity.^14^^,^^17^^,^^18^

Aerogels made of highly porous CNC represent an innovative class of renewable 3D matrices for SERS substrate fabrication,^9^^,^^19^ provided that an appropriate concentration of plasmonic nanoparticles is immobilized, homogeneously distributed through the entire matrix, and free to interact with the analyte. Moreover, in 3D structures the number of ‘hotspots’ available increase greatly in comparison with 1 and 2-dimensional substrates.^9^^,^^10^ This protocol describes the steps to fabricate SERS substrates using urea-choline chloride deep eutectic solvent and their usage for the detection of edifenphos and ethyl parathion in tea and rice extracts. These substrates may also be used for the detection of other analytes in various concentrations.

### Drying of choline chloride

**Timing: 24 h**

1. Before preparing the deep eutectic solvent, choline chloride (ChCl) should be dried to eliminate all traces of humidity in an oven, using an uncovered container at 75°C for 24 h.

### Choline chloride—Urea DES preparation

**Timing: 1.5 h**

2. Weigh the correct amount of choline chloride and urea at a 1:2 M ratio and mix them in a, preferably, transparent vial.

***Note:*** Although choline chloride – urea (ChCl-U) DES is highly stable for over a month, it is recommended to only prepare the exact amount, to avoid urea decomposition over time.

3. Mix them gently using a clean spatula, to aid the formation of the DES and then heat using a heating plate at 70°C for 1 h, with constant stirring with a magnetic bar at 500 rpm.

**CRITICAL:** To ensure the DES has formed as desired, there should be no traces of solids in the transparent, homogeneous DES.

***Note:*** To ensure heating takes place uniformly, all heating steps are done in an oil or sand bath.

***Note:*** DES can be used both hot, as prepared, or cold, however, volume measurements are more reliable when using warm DES, as its viscosity lessens.

## Key resources table

**Table:** 

REAGENT or RESOURCE	SOURCE	IDENTIFIER
**Chemicals, peptides, and recombinant proteins**		

Choline chloride ((2-hydroxyethyl)trimethylammonium chloride ≥98%)	Merck Millipore	CAS 67-48-1
Urea	Merck Millipore	CAS 57-13-6
Gold (III) chloride trihydrate (HauCl_4_⋅3H_2_O ≥ 99.9%)	Merck Millipore	CAS 16961-25-4
L-ascorbic acid	Merck Millipore	CAS 50-81-7
Cellulose nanocrystals (CNC)	CelluForce	N/A

**Other**		

50 mL Falcon tubes	Falcon	N/A
96-well cell culture plates	Corning	N/A
Thermo Scientific 8420-208200 UV-Vis spectrophotometer	Thermo Scientific	N/A
Senterra Raman spectrophotometer with 785 nm laser	Bruker	N/A
Freeze drier	Labconco	N/A
JEM1010 Transmission electron microscope	Jeol	N/A

## Materials and equipment

**Table:** 

Reagent	Final concentration	Amount	
Choline chloride	N/A	16.11 g	
Urea	N/A	13.72 g	
HAuCl_4_	2.05 mM	10 mg in 12.4 mL	
L-ascorbic acid	11.4 mM	25 mg in 12.4 mL	
CNC	0.5 wt%	75 mg in 12.4 mL	
**Total**	**N/A**	**24.8 mL**

***Note:*** The amount of choline chloride and urea were calculated based on the reagents listed in the key resources table and should be confirmed based on the reagents’ purity.

## Step-by-step method details

### Synthesis of gold nanoparticles onto cellulose nanocrystals

**Timing: 2.5 h**

**Figure 1:**
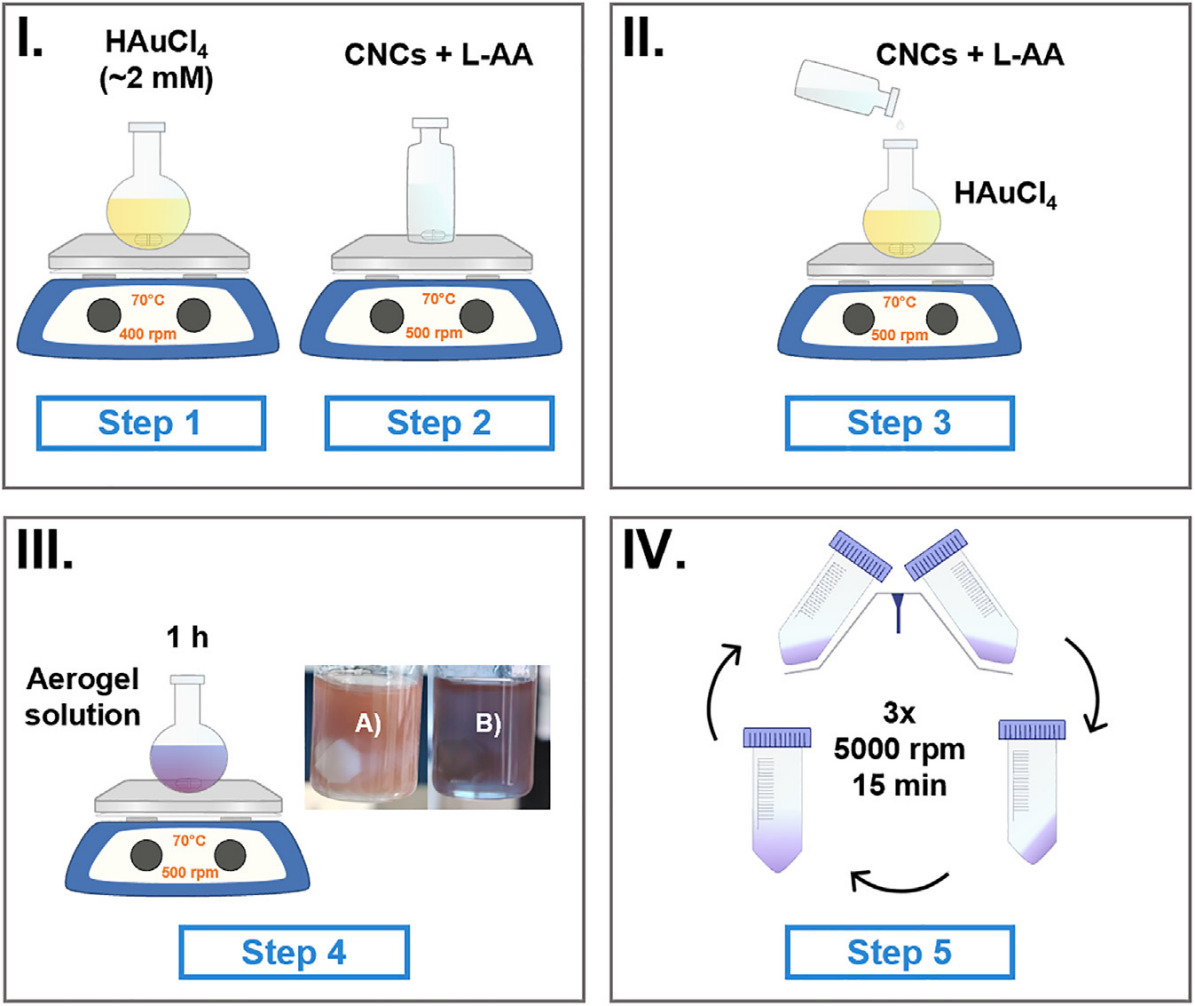
Schematic of the synthesis methodology for SERS aerogels (I) Preparation of the gold chloride and cellulose nanocrystals (CNCs) + L-ascorbic acid (L-AA) solutions in a ChCl-U DES. (II) Addition of the reductor (L-AA) to the HAuCl_4_ solution. (III) Reaction conditions and digital photographs of the obtained colloid coloration in A) reflection and B) transmission, both solutions have a magnetic stirrer. (IV) Washing steps to remove the DES.

This step describes the synthesis, washing steps and molding of the cellulose-gold substrates using a deep eutectic solvent (Figure 1).

1. Start with the preparation of the gold solution in a ChCl-U DES (2.05 mM) in a round bottom flask.
  a. Using a micropipette, deposit 12.4 mL of ChCl-U DES and 10 mg of HAuCl_4_ inside the flask.
  b. Sonicate the solution for 15 min at room temperature (25°C), to ensure the gold precursor has dissolved.
  c. Once dissolved, the mixture is heated at 70°C for 15 min with mild stirring (400 rpm).

**CRITICAL:** HAuCl_4_ is highly hydrophilic, low humidity or controlled atmosphere conditions are recommended to ensure there are no water traces.

***Note:*** Once the gold precursor is dissolved, the DES will acquire a bright yellow coloration.

**CRITICAL:** Using warm DES for dissolving HAuCl_4_ favors the formation of gold nanoparticles, it is recommended for a successful synthesis.

2. Prepare the CNC/L-ascorbic acid solution (3:1 w/w) in a round bottom flask.
  a. 25 mg of L-ascorbic acid (11.4 mM) are mixed with 75 mg (0.5 wt %) of CNC in 12.4 mL of DES.
  b. The mixture is stirred vigorously (900 rpm), followed by 10 min sonication at 25°C.
  c. Heat the CNC/L-ascorbic acid solution at 70°C for 15 min with constant stirring (500 rpm).
3. Add the CNC/L-ascorbic acid mixture to the gold precursor solution (See step 1), maintaining the temperature (70°C) and stirring (500 rpm) for 1 h.

**CRITICAL:** The L-ascorbic acid reduces the cellulose, precise timing while sonicating and heating are crucial (See Troubleshooting sec.).

**CRITICAL:** The synthesis is recommended to take place in tightly closed and dark conditions, as gold nanoparticles are photosensitive.

4. The reaction is stopped by quenching, adding equal parts of cold (10°C) deionized water (24.8 mL).
5. Immediately after, samples are washed in triplicate (3x) using centrifugation at 5000 rpm for 15 min at 25°C.
  a. Supernatant is removed and replaced with deionized water after each washing step, shaking vigorously with a vortex.
  b. The obtained final pellet is resuspended in 10 mL of deionized water (0.74 wt % of CNC) at 25°C.
6. The colloid is molded in 96-well cell culture plates, previously rinsed with 90% ethanol and left to dry.
  a. 100 μL of the colloidal solution are deposited in each well using a micropipette.
  b. The as-prepared wells are left to freeze at −12°C overnight, covered with parafilm.
7. Frozen samples are freeze-dried for 24 h at 12 Pa and −40°C.

***Note:*** Prepared aerogels should be stored in a desiccator, covered with parafilm, in order to avoid contamination or hydration.

**CRITICAL:** The colloid solution must be frozen overnight in a freezer, never below −20°C, as otherwise aerogels result brittle and hard to unmold. This is a result of the cellulose nanocrystals in colloidal state arranging themselves into a stable 3D network due to hydrogen bonding between the crystals during slow-freezing. On the other hand, when the substrates are frozen instantly (i.e. using liquid nitrogen), the crystals kinetic energy is reduced drastically, not allowing the network to form, thus leading to brittle aerogels.

### Preparation of rice and tea extracts for SERS measurements

**Timing: 1 h**

**Figure 2:**
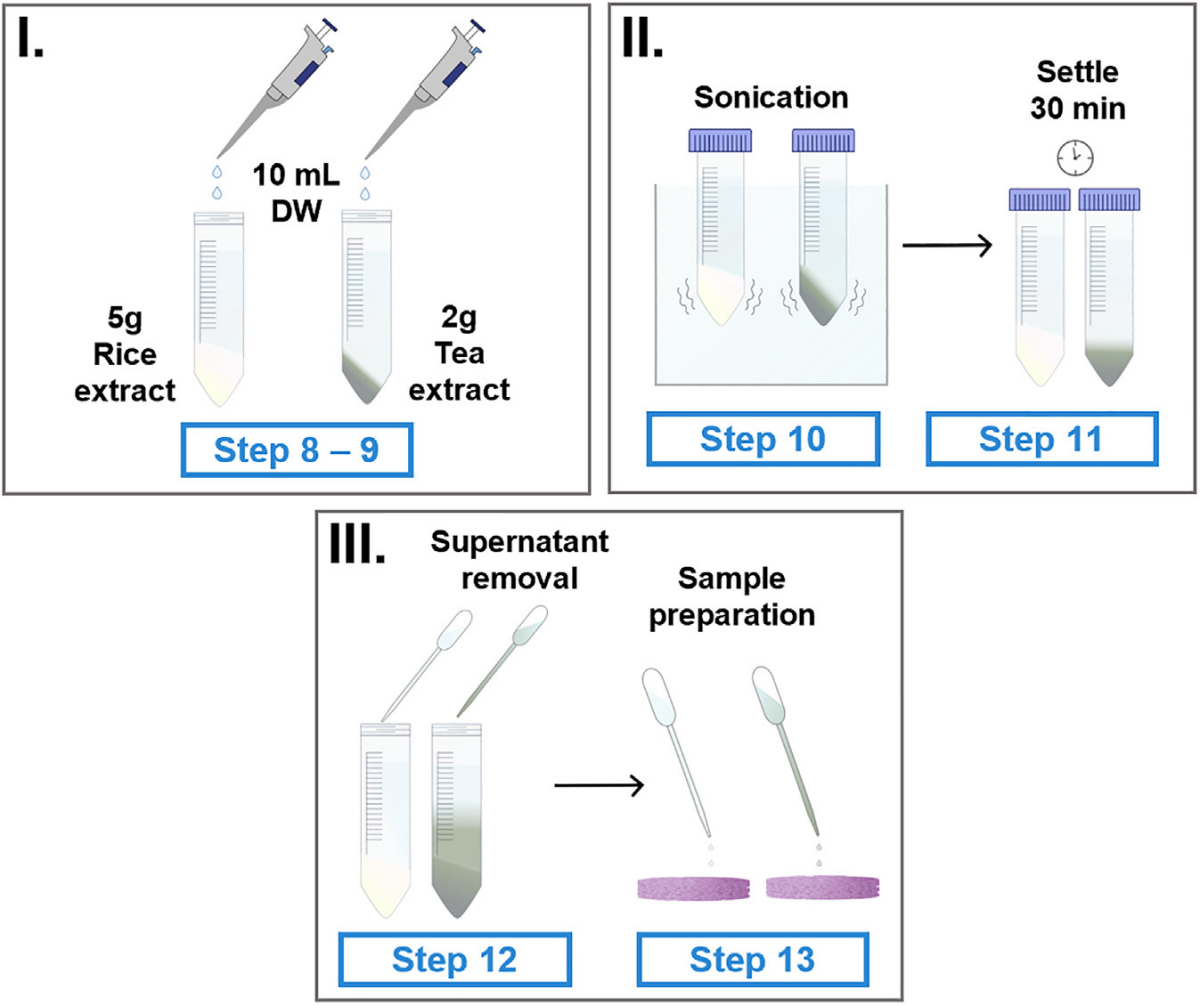
Schematic of the preparation of tea and rice extracts for SERS measurements (I) Addition of water to the dry samples. (II) Sonication of the samples for 5 and 20 min, rice and tea extracts respectively, followed by sedimentation. (III) The supernatant is removed and deposited onto the SERS substrates for Raman analysis.

This step describes the sample-preparation process for SERS measurements using the synthesized aerogels. It highlights key points for preparing the tea and rice extracts, as well as unmolding and loading samples into the aerogels (Figure 2).

8. Weigh the commercially purchased rice powder and black tea powder.
  a. Carefully weight 5 and 2 g of rice and tea powders, respectively, in clean falcon tubes.
9. Add 10 mL of deionized water (DW) to each of the falcon tubes containing the powders, using a micropipette.
10. Sonicate the rice and black tea mixtures for 5 and 20 min, respectively.
  a. Vigorously stirring the solutions using a vortex can also help ensure thorough mixing.
11. Allow the particles of both tubes to settle for 30 min.

**CRITICAL:** As the aerogels only collapse in the presence of deionized water and SERS is a sensitive technique, it is important to allow the particles to settle, in order not to have solid particles in the supernatant that could be transferred onto the substrates.

12. With the aid of a pipette, transfer the supernatant of both extractions to clean tubes.
  a. These extracts are kept in refrigeration (4°C) for up to a week.

**CRITICAL:** Is crucial to avoid disturbing the heavier particle residue at the bottom of the tubes.

13. Unmolding of the substrates onto glass slides and sample preparation.
  a. Each aerogel should be unmolded before use, with the aid of small tweezers, carefully detaching them from the plate by lifting from the center of the aerogel.
  b. Once unmolded, carefully place the aerogel onto the slide bottom-side up.
    **CRITICAL:** The synthesized aerogels are extremely light, take wind currents into consideration.
    ***Note:*** The aerogel is placed bottom-side up to ensure uniformity in sample deposition. During freeze-drying, due to the low volume of liquid in each well and the working pressure, the aerogel’s top tends to be irregular.
  c. Aerogels collapse with 15 μL of the analyte dispersed or diluted in deionized water.
  d. Drop the entire volume of the sample onto the aerogel with the aid of a micropipette.

14. Leave to dry at room temperature before measuring, to eliminate as much water as the tea and rice extract are used to prepare serial dilutions of edifenphos and ethyl parathion as required for further analysis or as described in our previously published work.^1^

**CRITICAL:** This sample preparation is simpler than the protocol required for inductively coupled plasma atomic emission spectroscopy (ICP-OES)

15. Raman spectra were obtained using a 785 nm laser with a 3 s integration time and 6 accumulations under a Bruker Senterra Raman microscope using a 1 mW laser power and a 20X objective.

## Expected outcomes

### Substrate characterization

**Figure 3:**
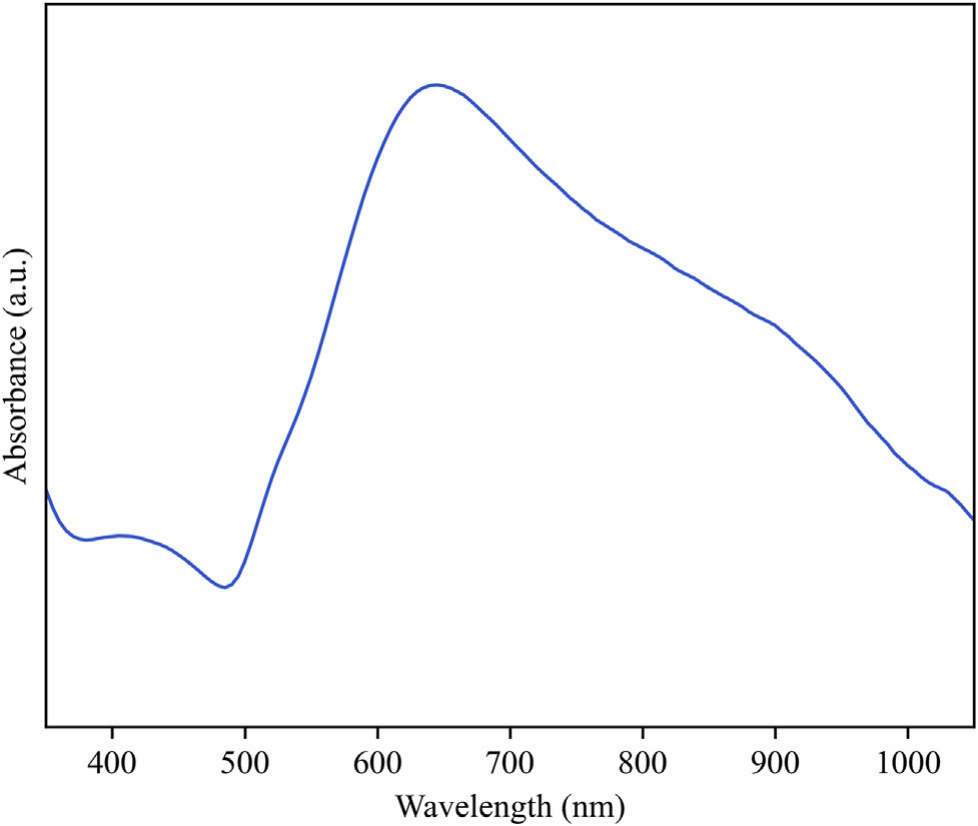
UV-Vis absorption spectra of the synthesized gold colloid

This protocol provides a synthesis methodology for the preparation of gold-decorated cellulose aerogels. This work is separated into important, clear steps to facilitate the reproduction of this methodology. The main outcome from this protocol is the formation of anisotropic gold nanoparticles deposited onto cellulose nanocrystals (CNCs), as SERS substrates. Herein we present the most significant results from the steps presented (Figure 3).

**Figure 4:**
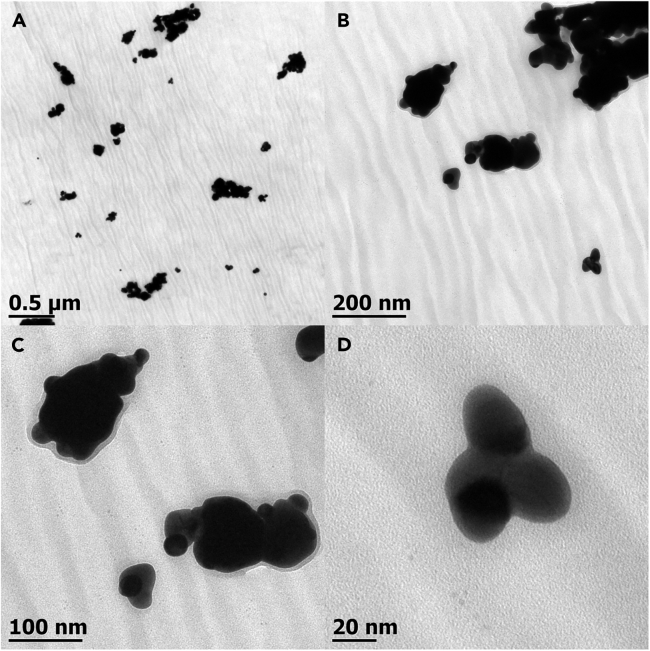
Transmission electron microscopy images of the obtained gold nanostructures at different magnifications Reproduced from Panikar et al.^1^

Figure 4 shows the UV-Vis spectra of the as-synthesized gold colloid, using 75 mg of cellulose nanocrystals. The plasmon resonance main excitation is located around 600 nm with a broad secondary plasmon excitation spanning from 750 to 900 nm. The presented absorption spectra are characteristic of the formed nanostructures, which plays a significant role in the electromagnetic field enhancement that promotes SERS.

**Figure 5:**
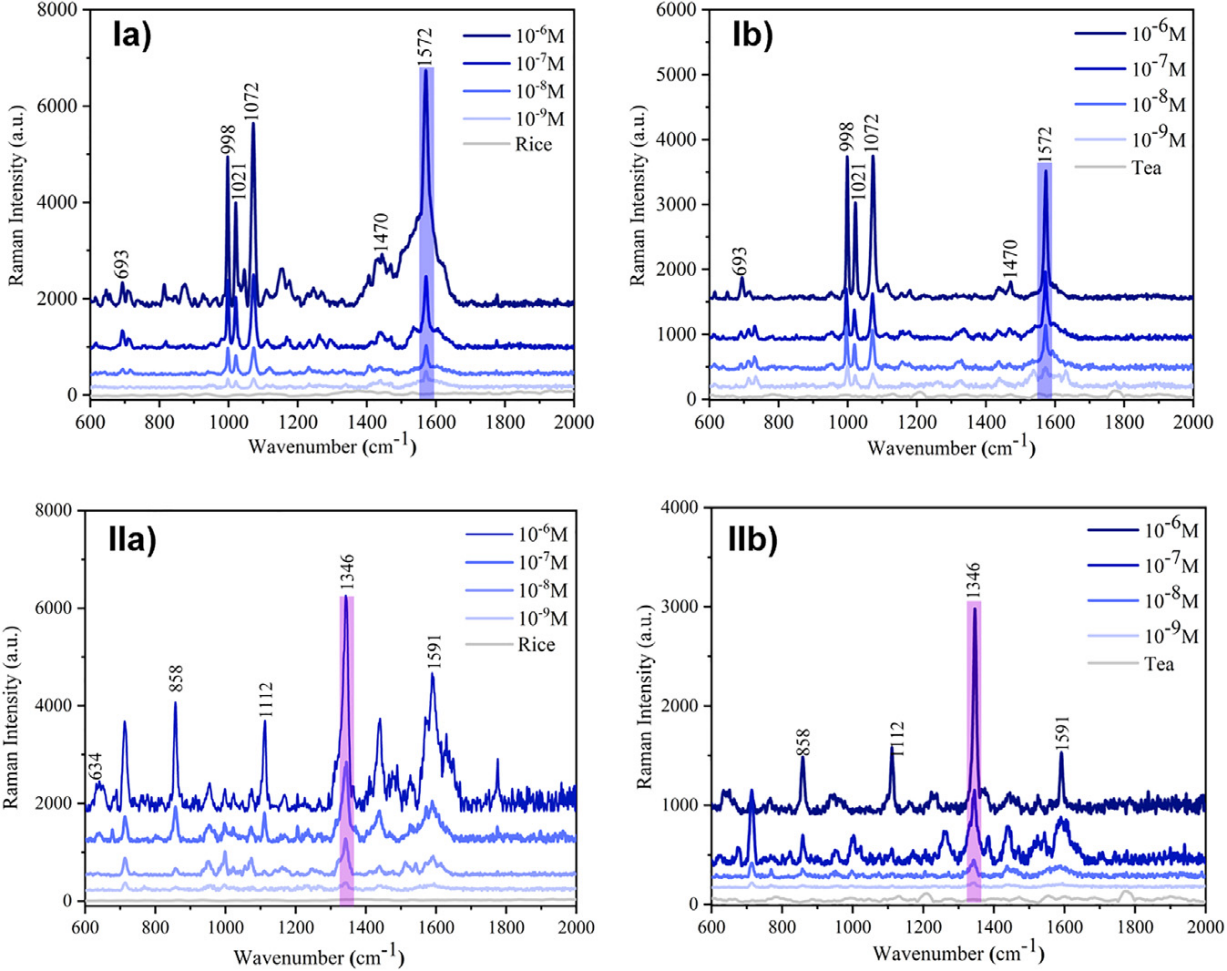
SERS spectra highlighting the characteristic vibration of each pesticide The spectra measure Edifenphos (I) and Ethyl Parathion (II) in rice (a) and tea (b) extracts. Reproduced from Panikar et al.^1^

Similarly, Figures 5A–5D shows TEM micrographs of the different nanostructures that can be obtained following this methodology. Both techniques are useful to corroborate the formation of the desired nanostructures.

These aerogels as SERS substrates have been utilized for the detection of diverse organophosphorus pesticides and several probe molecules.

### SERS performance of the synthesized substrates

The Surface-Enhanced Raman Scattering (SERS) enhancement factor (EF) is computed using the following equation:

$$\text{EF}=\frac{I_{\text{SERS}}/C_{\text{SERS}}}{I_{\text{NRS}}/C_{\text{NRS}}}$$

In this formula, I_SERS_ is the measured Raman intensity of the 4-aminothiophenol (4-ATP) molecules at a concentration (C_SERS_) of 10^−12^ M when adsorbed onto the 75-CNC aerogel substrate. I_NRS_ indicates the spontaneous Raman scattering intensity of the 4-ATP molecules at a concentration (C_NRS_) of 10^−6^ M when exposed to the laser on the CNC aerogel.^1^

Raman acquisition: At least five spectra were collected from three different plasmonic aerogel batches. The standard deviation represents the average of 30 spectra from these batches. The limit of detection (LOD) for analyte detection on the 75-CNC plasmonic aerogel was estimated by interpolating the intensity from deionized water-treated aerogel and averaging at least 30 spectra from three independently made substrates.

SERS validation: The CNC-based SERS substrates have demonstrated effectiveness in detecting the pesticides Edifenphos (EdP) and Ethyl Parathion (PRT) in complex matrices such as rice and tea extracts (Figure 5). While matrix effects result in higher detection limits compared to distilled water, the substrates maintain significant sensitivity. The limits of detection (LODs) for EdP are 8.72 nM in rice extract and 9.32 nM in tea extract, with a LOD of 10.38 pM in distilled water.^1^ For PRT, the LODs are 8.74 nM in rice extract and 8.6 nM in tea extract, while in distilled water, the LOD is 96.9 pM.^1^

Importance: These findings highlight the substrates' potential for practical applications in agricultural pesticide monitoring and environmental analysis, demonstrating reliable detection capabilities even within complex sample matrices.

## Limitations

Despite the straightforward synthesis and reproducibility of the methodology, ambient conditions, mainly humidity, play a crucial role in the preparation of the materials, synthesis and formation of the aerogels and sample preparation for SERS measurements. This is because the metallic precursor, choline chloride and the prepared aerogels are highly hydrophilic, which leads to variations in particle size, morphology and the capacity of the aerogels to collapse. It is also worth noting that aerogels only collapse in presence of water, analytes in nonaqueous media cannot be measured using these substrates.

## Troubleshooting

All reagents and materials used in this protocol can be substituted by reagents purchased from other suppliers; we do strongly suggest utilizing the reagents mentioned in the ‘key resources table’ to ensure reproducibility.

### Problem 1

Preparation of the gold solution in DES from HAuCl_4_ trihydrate (See step 1) due to hydration of the precursor leads to varying results in terms of morphology, size distribution and concentration of the nanoparticles, as well as stability of the aerogels and SERS response of the substrates.

***Note:*** The utilized DES (ChCl-U) stops hydration of the gold precursor, as there is no water content it leads to precise control over the gold concentration.

### Potential solution

• Use a stock solution: Instead of preparing the gold precursor by directly adding the solid to DES, prepare a highly concentrated stock solution in water, so that the volume added to DES does not surpass 2 wt % of the total mass. This way the DES’s composition is not affected by the slight presence of water and the gold concentration is achieved with precision.
• Cleanroom: If such facility is available at your institution, it will allow the precise weighing of the precursor and preparation of the precursor solution in DESs.

### Problem 2

Incorrect dispersion of CNC into the deep eutectic solvent (See step 2). Due to the choline chloride-urea DESs viscosity, dispersing the cellulose crystals and L-ascorbic acid can be problematic. The heterogeneity of the reducing solution leads to different nanoparticle morphologies and, thus, variations in the SERS measurements.

### Potential solution

• Sonicate the solution for longer time with heating: Instead of simply sonicating, add temperature while sonicating (∼35°C), this will reduce the DESs viscosity without risking degradation of L-ascorbic acid.

**CRITICAL:** The sonicating and heating steps should not surpass 30 min. The L-ascorbic acid reduces the CNCs during these steps, this reduction favors the interaction between CNCs and the as-formed gold nanoparticles; however, excessive reduction leads to detrimental effects, such as poor interactions with the nanostructures and even the hindering of the reaction, which could prevent the formation of nanoparticles.

• Alternate between sonicating and stirring with a magnetic stirrer: CNCs tend to form lumps inside the DES, which are difficult to break apart with sonication, stirring will help break the lumps to facilitate the dispersion of the crystals in DES.
• Heat the DES before attempting to disperse CNCs: As previously mentioned, the viscosity of DESs decreases with increasing temperature, pre-heating the DES at 50°C will facilitate its manipulation and the dispersion of CNC and L-ascorbic acid in it.

**CRITICAL:** Do not overheat DES, for longer than 20 min, nor heat above 75°C, as the urea in the DES can start to decompose, which results in variations of morphology and size of the synthesized nanostructures.

## Resource availability

### Lead contact

Further information and requests for resources and reagents should be directed to and will be fulfilled by the lead contact, Josué D. Mota-Morales (jmota@fata.unam.mx).

### Technical contact

Technical questions on executing this protocol should be directed to and will be answered by the technical contact, Kaori Sánchez-Carrillo (kaoris15@comunidad.unam.mx).

### Materials availability

Not available.

### Data and code availability

No new datasets were generated.

## Acknowledgments

This work was supported by SECIHTI (formerly CONAHCYT) through Project CF-2023-I-1339 and PAPIIT-UNAM Project IN115624.

All illustrations are of K.S.-C.’s authorship.

## Author contributions

K.S.-C.: validation and writing – original draft; S.S.P.: methodology, investigation, and formal analysis; and J.D.M.-M.: conceptualization, writing – review and editing, and supervision.

## Declaration of interests

The authors declare no competing interests.
